# Characterization of Atrasentan Metabolic Pathway in Human Liver Microsomes Using Feature-Based Molecular Networking

**DOI:** 10.3390/pharmaceutics18060731

**Published:** 2026-06-13

**Authors:** Hyung-Ju Seo, Zhuoning Liang, Eui-Hyeon Kim, Kwang-Hyeon Liu

**Affiliations:** 1 BK21 FOUR KNU Community-Based Intelligent Novel Drug Discovery Education Unit, College of Pharmacy, Research Institute of Pharmaceutical Sciences, Kyungpook National University, Daegu 41566, Republic of Korea; hlhl103@naver.com (H.-J.S.); lzn0430@gmail.com (Z.L.); 2 Clinical Omics Institute, School of Medicine, Kyungpook National University, Daegu 41405, Republic of Korea; 3 Mass Spectrometry Based Convergence Research Institute, Kyungpook National University, Daegu 41566, Republic of Korea

**Keywords:** atrasentan, characterization, drug-induced liver injury, feature-based molecular networking, metabolism, reactive metabolite

## Abstract

**Background/Objectives**: Atrasentan is a selective endothelin A receptor antagonist (SERA) developed as a potential therapy for chronic renal diseases, including diabetic nephropathy and immunoglobulin A nephropathy. Despite this potential, understanding its metabolic bioactivation is essential for assessing the risks of drug-induced liver injury (DILI). However, the metabolic profile of atrasentan remains poorly characterized, and the mechanisms underlying its potential hepatotoxicity remain underexplored. Therefore, this study aims to investigate the metabolic pathways of atrasentan in human liver microsomes (HLMs) in the presence of nicotinamide adenine dinucleotide phosphate (NADP^+^), uridine diphosphate glucuronic acid (UDPGA), or glutathione (GSH). **Methods**: A liquid chromatography–high resolution mass spectrometry (LC-HRMS) coupled with a feature-based molecular networking approach was used to characterize metabolites. Characterization of the major metabolites was achieved through cytochrome P450 (P450) phenotyping with human recombinant P450 isoforms. **Results**: A total of eighteen metabolites were characterized through phase I and II metabolic reactions, including demethylenation, *N*-dealkylation, *O*-demethylation, hydroxylation, dehydrogenation, and glucuronidation. Atrasentan acyl glucuronide (**M8**) was confirmed as the predominant metabolite, and we also putatively annotated a catechol intermediate (**M5**) and its corresponding GSH conjugate (**M15**). Characterizing the GSH conjugate (**M15**) indicates that catechol intermediate (**M5**) can be further oxidized to a reactive *ortho*-quinone intermediate, which is subsequently trapped by GSH, suggesting the potential for a bioactivation mechanism. Reaction phenotyping demonstrated that the formation of **M5** is catalyzed almost exclusively by the CYP3A subfamily. However, its direct translation to in vivo oxidative stress or covalent protein binding requires further studies. **Conclusions**: These findings demonstrate that feature-based molecular networking is a valuable strategy for metabolite characterization, underscoring the urgent need for further in vivo metabolism studies to definitively assess hepatotoxic risks associated with these reactive metabolites.

## 1. Introduction

Atrasentan, a selective endothelin A receptor antagonist (SERA), is a promising therapeutic candidate for the management of chronic kidney disease, demonstrating efficacy in diabetic nephropathy and immunoglobulin A nephropathy [1,2]. By attenuating the deleterious effects of endothelin-1, atrasentan helps preserve renal function and reduce proteinuria [3]. Despite this significant therapeutic potential, the clinical advancement of endothelin receptor antagonists has been historically limited due to safety concerns, particularly the risk of drug-induced liver injury (DILI) [3,4]. Consequently, a comprehensive evaluation of the hepatotoxic potential of atrasentan is essential to ensure its long-term clinical safety.

**Figure 1:**
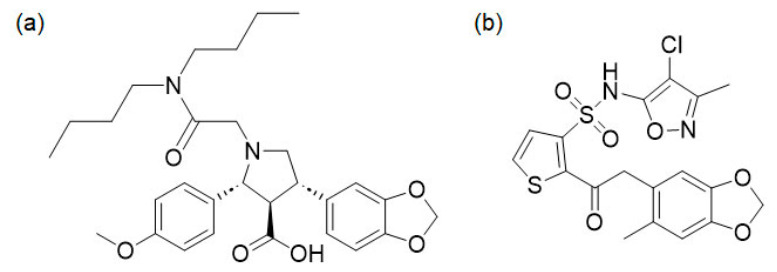
Chemical structure of (**a**) atrasentan and (**b**) sitaxentan.

This safety concern is not merely theoretical but is underscored by the clinical history of sitaxentan (Figure 1), a structurally related SERA in the same pharmacological class [4,5]. Originally approved for pulmonary arterial hypertension, sitaxentan was voluntarily withdrawn from the global market in 2010 after reports of severe idiosyncratic hepatotoxicity, leading to acute liver failure and fatal outcomes [6]. Subsequent mechanistic studies revealed sitaxentan-induced hepatotoxicity to metabolic bioactivation of its methylenedioxyphenyl moiety [7]. This structure undergoes cytochrome P450 (P450)-mediated oxidation and demethylenation, forming an unstable catechol intermediate that is oxidized further into a highly reactive electrophilic *ortho*-quinone [8]. This reactive intermediate can deplete intracellular glutathione (GSH) and covalently bind to nucleophilic sites on critical cellular proteins, forming drug-protein adducts [9,10].

Similar to sitaxentan, atrasentan contains a methylenedioxyphenyl moiety in its scaffold, raising concerns about potential bioactivation and hepatotoxicity [11]. In current clinical trials, atrasentan demonstrates a favorable safety profile, but the precise metabolic fate of this structural motif remains incompletely characterized [12]. In particular, whether the methylenedioxyphenyl ring of atrasentan undergoes bioactivation to form reactive *ortho*-quinone species remains unclear [4].

Therefore, this study aims to investigate the metabolic pathways of atrasentan using human liver microsomes (HLMs) [4,13]. Incubations were performed with nicotinamide adenine dinucleotide phosphate (NADPH) and uridine diphosphate glucuronic acid (UDPGA) to phase I and II metabolite profiling [13,14]. Furthermore, building on toxicological insights from sitaxentan, we employed a GSH-trapping strategy to sequester and stabilize transient, short-lived electrophilic intermediates. High-resolution mass spectrometry (HRMS) coupled with feature-based molecular networking (FBMN) was used for precise metabolite characterization [15]. FBMN utilizes MS/MS spectral similarity (evaluated via cosine scores) to align and connect structurally related metabolites into a molecular network. Each node within the network represents a consensus MS/MS spectrum of a specific metabolite feature, while the connecting edges denote structural similarities calculated from matching fragment ions and neutral losses [16,17]. This allows for the comprehensive visualization of structural relationships between atrasentan and its metabolites. Therefore, this approach provides a useful tool for prioritizing candidate metabolites and exploring the potential bioactivation pathways of atrasentan.

## 2. Materials and Methods

### 2.1. Chemicals and Reagents

We sourced atrasentan, along with essential cofactors and reagents including glutathione (GSH), glucose-6-phosphate (G6P), glucose-6-phosphate dehydrogenase (G6PDH), nicotinamide adenine dinucleotide phosphate (NADP^+^), magnesium chloride (MgCl_2_), and uridine diphosphate glucuronic acid (UDPGA), directly from Sigma-Aldrich (St. Louis, MO, USA). Toronto Research Chemicals (Toronto, ON, Canada) provided the alamethicin. HLMs (pooled, mixed-gender, Xtreme 200, H2630) were procured through XenoTech (Lenexa, KS, USA). Human recombinant P450 isoforms (rCYP1A2, 2A6, 2B6, 2C8, 2C9, 2C19, 2D6, 2E1, 3A4, and 3A5) were supplied by SPMED (Busan, Republic of Korea). Any additional chemicals and solvents utilized in this study met LC-MS grade specifications and were acquired from Fisher Scientific (Pittsburgh, PA, USA).

### 2.2. Characterization of Phase I and II Metabolites in Human Liver Microsomes

Metabolite profiling was conducted following previously established protocols with minor modifications [15]. After dissolving the drug in dimethyl sulfoxide to make a stock, 100 µL incubation mixtures comprised 100 µM atrasentan, 1 mg/mL of HLMs, and 100 mM potassium phosphate buffer (pH 7.4).

For phase I metabolism, a cofactor mixture acting as the NADPH-generating system (1.3 mM NADP^+^, 3.3 mM G6P, 1 U/mL G6PDH, and 3.3 mM MgCl_2_) was incubated with samples at 37 °C for 2 h.

For phase II metabolism, 5 mM UDPGA and 25 µg/mL alamethicin were added. Alamethicin was used to permeabilize membranes, thereby enabling adequate UDPGA access to the enzymatically active sites for the glucuronidation of atrasentan and its metabolites [18]. The addition of cold acetonitrile in a 1:1 ratio effectively stopped the reactions. To precipitate the proteins, the mixtures were centrifuged at 12,700 rpm (4 °C) for 10 min. The supernatants were then filtered through 0.2 µm polyvinylidene fluoride (PVDF) membranes before the LC-HRMS system. All data points were collected in triplicate, including control samples containing microsomes deactivated by heating at 100 °C for 30 min.

### 2.3. Characterization of Reactive Metabolites in Human Liver Microsomes

To characterize reactive intermediate formation, 100 µM atrasentan and 1 mg/mL HLMs were mixed in a 100 mM potassium phosphate buffer (pH 7.4). This mixture was enriched with 5 mM GSH acting as a trapping agent, along with an NADPH-generating system, and maintained at 37 °C for 2 h. The addition of cold acetonitrile in a 1:1 ratio effectively quenched the reactions. To precipitate the proteins, the mixtures were centrifuged at 12,700 rpm (4 °C) for 10 min. The supernatants were then filtered through 0.2 µm PVDF membranes before LC-HRMS analysis. All experiments were executed in triplicate, including negative controls employing microsomes inactivated by heat treatment at 100 °C for 30 min.

### 2.4. Reaction Phenotyping of Atrasentan in Human Recombinant P450 Isoforms

Preliminary kinetic assays utilizing HLMs were conducted to optimize incubation conditions and ensure reproducibility. The generation of the three major metabolites (**M2**, **M3**, and **M5**) demonstrated linearity over a 60 min incubation window at substrate concentrations of both 10 and 50 µM. Consequently, a 30 min incubation period was selected for subsequent profiling experiments. The minimal variation in metabolic rates across replicates validated the HLM system.

For reaction phenotyping, 50 µM atrasentan was incubated with 50 pmol/mL human recombinant P450 isoforms (rCYP1A2, rCYP2A6, rCYP2B6, rCYP2C8, rCYP2C9, rCYP2C19, rCYP2D6, rCYP2E1, rCYP3A4, and rCYP3A5) in 100 mM potassium phosphate buffer (pH 7.4). A cofactor mixture serving as the NADPH-generating system was introduced, and the samples were incubated at 37 °C for 30 min. The reactions were terminated by the addition of cold acetonitrile in a 1:1 ratio. To precipitate the proteins, the mixtures were centrifuged at 12,700 rpm (4 °C) for 10 min. The supernatants were then filtered through 0.2 µm PVDF membranes prior to LC-MS/MS analysis. All data points were collected in independent triplicate, including control samples containing microsomes deactivated by heating at 100 °C for 30 min.

### 2.5. LC–MS/MS Conditions

Metabolite characterization was executed on an UltiMate 3000 HPLC system interfaced with a Q Exactive Focus Orbitrap HRMS (Thermo Fisher Scientific, Waltham, MA, USA). To achieve chromatographic resolution, samples were injected onto an Acquity UPLC HSS C18 column (100 × 2.1 mm, 1.8 µm, 100 Å; Waters, Milford, MA, USA) maintained at a steady 40 °C. The mobile phase comprised 0.1% formic acid in water (A) and 0.1% formic acid in acetonitrile (B), running at 0.2 mL/min with a 2 µL injection volume. The linear gradient program was set as: 20–40% B (0–5 min), 40–90% B (5–10 min), 90–20% B (10–10.1 min), and 20% B (10.1–13 min).

Ionization was performed via positive electrospray ionization (ESI) under the following optimized settings: spray voltage, 3.0 kV; capillary temperature, 320 °C; S-lens RF level, 50.0 V; sheath gas, 35 arbitrary units; and auxiliary gas, 10 arbitrary units. Mass spectra were captured utilizing a full-scan mode (*m*/*z* 300–900 range, 70,000 mass resolution, 1 × 10^6^ AGC target) concurrently with data-dependent tandem mass spectrometry (ddMS^2^). For the ddMS^2^ events, the system isolated the top three abundant precursors per cycle, applying a normalized collision energy of 30 eV at a resolution of 35,000. Instrument control and subsequent data handling were governed by Xcalibur software version 4.7.

Reaction phenotyping was conducted using on a Shimadzu 8060 triple quadrupole LC-MS/MS system (Shimadzu, Kyoto, Japan). The chromatographic conditions, including the analytical column, mobile phase composition, and gradient elution program, were kept identical to those described above for the HRMS analysis. Detection was managed via multiple reaction monitoring (MRM) in ESI positive mode. The specific MRM transitions were tracked at a collision energy of 25 eV: *m*/*z* 455.22 → 354.13 (**M2**), *m*/*z* 497.26 → 340.12 (**M3**), and *m*/*z* 499.28 → 342.13 (**M5**).

### 2.6. Profiling of Atrasentan Metabolites Using Feature-Based Molecular Network (FBMN)

To process the raw LC-HRMS datasets, RAW files were converted into the mzML format using MSConvert 3.0 (ProteoWizard; https://proteowizard.sourceforge.io, accessed on 19 November 2025). The mzML files were subsequently imported into MZmine 4.0 for feature detection, chromatogram building, deconvolution, and isotopic grouping, in accordance with previously optimized parameters [15]. The feature quantification (.csv) and spectral (.mgf) files were exported for molecular networking. The resulting feature quantification (.csv) and spectral (.mgf) files were exported and uploaded to the global natural products social molecular networking (GNPS) web platform via the WinSCP 6.5 client (https://winscp.net, accessed on 19 November 2025).

Molecular networking was executed using the FBMN tool hosted on the GNPS website (http://gnps.ucsd.edu, accessed on 19 November 2025) [15]. Mass tolerance boundaries were defined at 0.02 Da for both precursor and fragment ions. Network edges were established by applying a cosine similarity threshold of at least 0.6, requiring a minimum of five matching fragment peaks; alternatively, edges were formed if the paired nodes ranked within each other’s top 10 most related neighbors. The finalized network topology was visualized and analyzed using Cytoscape software version 3.1 (https://cytoscape.org, accessed on 19 November 2025) [19,20].

## 3. Results and Discussion

Standard solutions of atrasentan were introduced into the mass spectrometer via ESI in positive and negative modes to determine the optimal ionization polarity. The positive ionization mode produced significantly higher signal intensity than that of the negative mode. Given that atrasentan possesses basic nitrogen atoms capable of protonation, the positive mode was used for all subsequent analyses, consistent with previous report [15].

To verify experimental reproducibility, all HLM incubations were performed in independent triplicates. The detection of all eighteen metabolites, including the GSH conjugate, was consistent across all replicates, with the acceptable relative standard deviations (RSDs) of the extracted ion chromatogram (EIC) peak areas for the predominant metabolites (<15%).

### 3.1. Characterization of Atrasentan Phase I Metabolites

Metabolite structures were characterized based on accurate mass measurements with mass errors within 5 ppm and characteristic fragment ion patterns, using the fragmentation pattern of the atrasentan as a reference.

**Figure 2:**
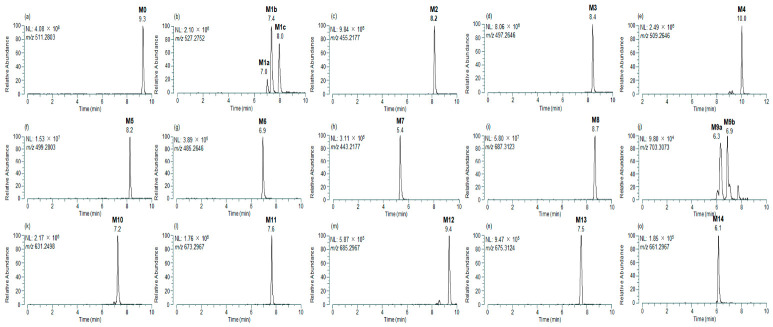
Representative extracted ion chromatograms of (**a**) atrasentan and (**b**–**o**) its metabolites generated from the liquid chromatography–high resolution mass spectrometry analysis of human liver microsomal incubation samples.

**Figure 3:**
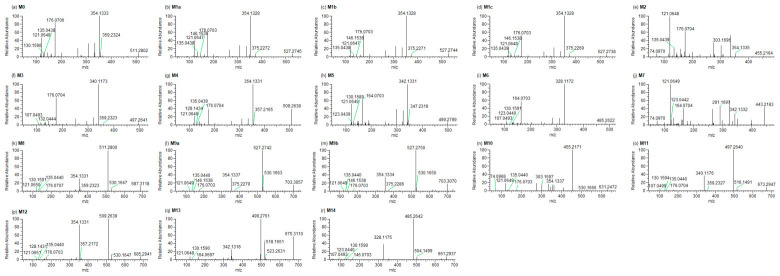
Representative product ion scan mass spectra of (**a**) atrasentan and (**b**–**r**) its metabolites. Table 1 provides detailed information on each metabolite.

**Figure 4:**
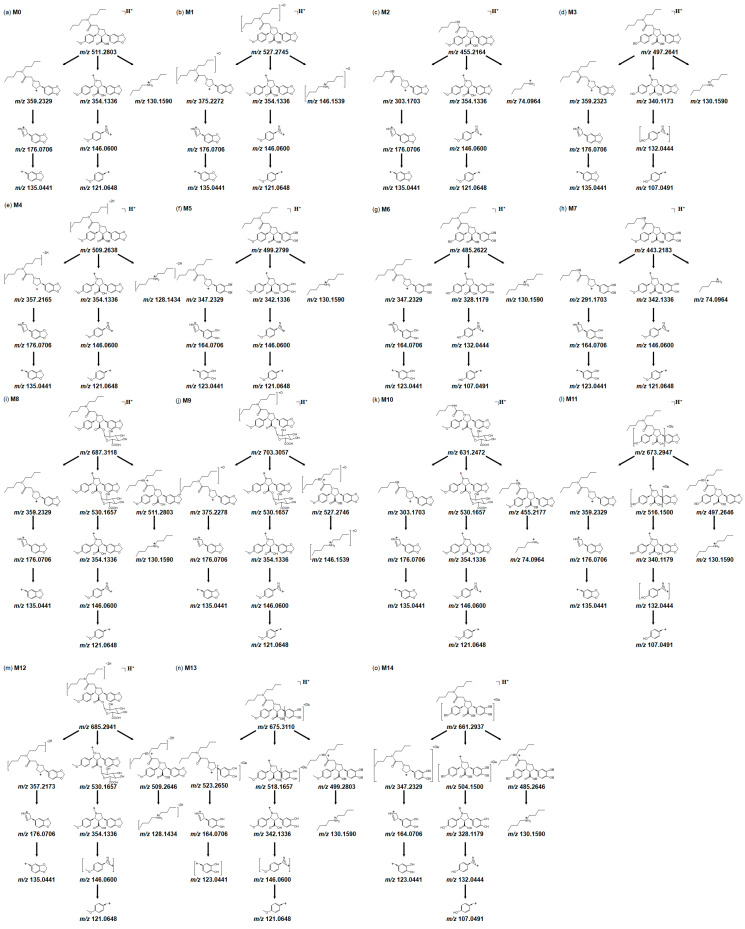
Representative MS/MS fragmentation schemes for (**a**) atrasentan and (**b**–**o**) its metabolites. Table 1 provides the corresponding detailed information.

The protonated molecular ion of atrasentan (**M0**) was observed at *m*/*z* 511.2802 (C_29_H_39_N_2_O_6_) and eluted at 9.3 min (Figure 2a). Its MS/MS spectrum showed fragment ions at *m*/*z* 359.2324, 354.1333, 176.0706, 130.1590 and 121.0649 (Figure 3a). The fragment ion at *m*/*z* 359.2324 resulted from the loss of the methoxyphenyl moiety, *m*/*z* 176.0706 represented the pyrrolidine fused with a methylenedioxyphenyl moiety. Additionally, *m*/*z* 130.1590 was characterized as the dibutylamine moiety, forming a complementary pair with the fragment ion at *m*/*z* 354.1333, and *m*/*z* 121.0649 corresponded to the methoxyphenyl moiety (Figure 4a). These fragments support the structural characterization of the metabolites (Table 1).

In total, nine phase I metabolites (**M1**–**M7**) were annotated in the human liver microsomal incubation samples. Metabolites **M1a**, **M1b**, and **M1c** eluted at 7.0, 7.4, and 8.0 min, respectively (Figure 2b), each exhibiting a protonated molecular ion at *m*/*z* 527.2738–527.2745 (C_29_H_39_N_2_O_7_). The observed mass shift of **+16 Da** relative to **M0** suggests the incorporation of an oxygen atom, consistent with a hydroxylation modification. The MS/MS spectrum revealed that the characteristic fragment ions at *m*/*z* 121.0647 and 176.0703 remained unchanged, suggesting that the methoxyphenyl and methylenedioxyphenyl-pyrrolidine moieties were intact (Figure 3b–d). In contrast, the fragment ions observed at *m*/*z* 359.2324 and 130.1590 in **M0** exhibited a **+16 Da** shift to *m*/*z* 375.2272 and 146.1539 (Figure 4b), respectively, indicating that the hydroxylation occurred on the dibutylamine moiety. However, the precise position of the hydroxyl group within the butyl chains could not be definitively assigned due to the lack of informative fragment ions to distinguish between positional isomers.

Metabolite **M2** eluted at 8.2 min (Figure 2c) and exhibited a protonated molecular ion at *m*/*z* 455.2164 (C_25_H_31_N_2_O_6_). The observed mass shift of **−56 Da** relative to **M0** suggests the loss of a butyl group (C_4_H_8_), consistent with an *N*-dealkylation modification. In the MS/MS spectrum of **M2**, the diagnostic fragment ions at *m*/*z* 359.2324 and 130.1590 observed in **M0** were replaced by *m*/*z* 303.1696 and 74.0970, consistent with the loss of a butyl chain from the amine moiety (Figure 3e). Conversely, the fragment ions at *m*/*z* 176.0704 and 121.0648 were retained, indicating that the core ring systems remained unaltered (Figure 4c). This pattern localized the *N*-dealkylation to the dibutylamino chain.

Metabolite **M3** eluted at 8.4 min (Figure 2d) and exhibited a protonated molecular ion at *m*/*z* 497.2641 (C_28_H_37_N_2_O_6_). The observed mass shift of **−14 Da** relative to **M0** suggests the loss of a methyl group (CH_2_), consistent with an *O*-demethylation modification. In the MS/MS spectrum of **M3**, the diagnostic fragment ions at *m*/*z* 354.1333 and 121.0649 observed in **M0** were replaced by *m*/*z* 340.1173 and 107.0493, consistent with the loss of a methyl group from the methoxyphenyl moiety (Figure 3f). Conversely, the fragment ion at *m*/*z* 359.2323 was retained, indicating that the butyl chains and methylenedioxyphenyl moieties remained unmodified (Figure 4d). This pattern localized the *O*-demethylation to the methoxyphenyl group.

Metabolite **M4** eluted at 10.0 min (Figure 2e) and exhibited a protonated molecular ion at *m*/*z* 509.2638 (C_29_H_37_N_2_O_6_). The observed mass shift of **−2 Da** relative to **M0** suggests the loss of two hydrogen atoms, consistent with a dehydrogenation modification. This retention behavior is consistent with the formation of a double bond in the alkyl chain, which maintains lipophilicity and leads to elution after the parent compound [21,22]. In the MS/MS spectrum of **M4**, the fragment ion at *m*/*z* 130.1590 observed in **M0** was replaced by *m*/*z* 128.1434 (Figure 3g), while the primary core fragment shifted from *m*/*z* 359.2324 to 357.2165 (Figure 4e). These changes localized the dehydrogenation to the dibutylamino chain. Although the formation of a double bond in the alkyl chain was confirmed, its exact location could not be determined based solely on the MS/MS fragmentation pattern.

Metabolite **M5** eluted at 8.2 min (Figure 2f) and exhibited a protonated molecular ion at *m*/*z* 499.2799 (C_28_H_39_N_2_O_6_). The observed mass shift of **−12 Da** relative to **M0** suggests the loss of a carbon atom, consistent with a demethylenation modification of the methylenedioxyphenyl group, leading to the formation of a catechol intermediate (Figure 3h). In the MS/MS spectrum of **M5**, the fragment ions at *m*/*z* 359.2324 and 176.0706 observed in **M0** were replaced by *m*/*z* 347.2318 and 164.0706, corresponding to the catechol-pyrrolidine moiety (Figure 4f). Concurrently, the detection of unmodified product ions at *m*/*z* 130.1598 and 121.0648 confirmed that the dibutylamine and methoxyphenyl groups remained intact. This pattern localized the demethylenation to the formation of a catechol intermediate (Table 1). Among the phase I metabolites, M5 was detected as a major metabolite under the current incubation conditions. Since catechol intermediate can undergo oxidation to form electrophilic *ortho*-quinones, this suggests that the metabolic route leading to the formation of reactive intermediates warrants closer evaluation [23].

Metabolite **M6** eluted at 6.9 min (Figure 2g) and exhibited a protonated molecular ion at *m*/*z* 485.2622 (C_27_H_37_N_2_O_6_). The observed mass shift of **−26 Da** suggests the combined loss of a methyl group and a carbon atom, consistent with concurrent demethylenation and *O*-demethylation (Figure 3i). MS/MS fragmentation revealed that the fragment ion at *m*/*z* 121.0649 shifted to 107.0493, corresponding to the *O*-demethylation of the methoxyphenyl moiety, while *m*/*z* 176.0706 shifted to 164.0703, consistent with demethylenation to a catechol moiety (Figure 4g). Thus, **M6** was characterized as the product of concurrent demethylenation and *O*-demethylation (Table 1).

Metabolite **M7** eluted at 5.4 min (Figure 2h) and exhibited a protonated molecular ion at *m*/*z* 443.2183 (C_24_H_31_N_2_O_6_). The observed mass shift of **−68 Da** relative to **M0** suggests the combined loss of a butyl group and a carbon atom, consistent with concurrent demethylenation and *N*-dealkylation (Figure 3j). The fragment ion at *m*/*z* 359.2324 shifted to *m*/*z* 291.1687, consistent with the simultaneous loss of a butyl chain and demethylenation to a catechol moiety (Figure 4h). The fragment ion at *m*/*z* 121.0649 remained preserved, confirming an unmodified methoxyphenyl group (Table 1).

### 3.2. Characterization of Atrasentan Phase II Metabolites

In total, eight phase II metabolites (**M8M14**) were detected, resulting from the glucuronidation of atrasentan (**M0**) or its phase I metabolites.

Metabolite **M8** eluted at 8.7 min (Figure 2i) and exhibited a protonated molecular ion at *m*/*z* 687.3118 (C_35_H_47_N_2_O_12_). The observed mass shift of **+176 Da** relative to **M0**, combined with the presence of a carboxylic acid group in the parent drug, suggests the conjugation of a glucuronic acid moiety (C_6_H_8_O_6_), indicating acyl glucuronidation [24]. In the MS/MS spectrum, the protonated molecular ion generated a major fragment ion at *m*/*z* 511.2800 via the neutral loss of **176 Da**, corresponding to the protonated **M0** (Figure 3k). The fragment ion at *m*/*z* 530.1666, which corresponds to the loss of the dibutylamine moiety while retaining the intact glucuronic acid, clearly suggested that the glucuronic acid was conjugated to the acyl moiety, providing evidence for acyl glucuronidation. The subsequent fragmentation pattern was identical to that of **M0**, exhibiting characteristic ions at *m*/*z* 359.2331 and 176.0707 (Figure 4i). This specific mass shift and fragmentation pattern support **M8** as the acyl glucuronide of **M0** (Table 1). Among all atrasentan metabolites, **M8** was the most predominant.

Metabolites **M9a** and **M9b** eluted at 6.3 and 6.9 min, respectively (Figure 2j), and exhibited protonated molecular ions at *m*/*z* 703.3057–703.3070 (C_35_H_47_N_2_O_13_). The observed mass shift of **+192 Da** relative to **M0** corresponds to the combination of hydroxylation (**+16 Da**) and glucuronidation (**+176 Da**). The MS/MS spectra exhibited a characteristic neutral loss of **176 Da**, producing a predominant fragment ion at *m*/*z* 527.2742, corresponding to the protonated ion of the hydroxylated metabolite **M1** (Figure 3m). Notably, the detection of the diagnostic fragment ion at *m*/*z* 530.1663, which retains the intact glucuronic acid conjugated to the acyl moiety after the loss of the hydroxylated dibutylamine moiety, providing evidence for acyl glucuronidation. Subsequent fragmentation of the *m*/*z* 527.2742 ion yielded diagnostic ions at *m*/*z* 375.2269 and 146.1538 (Figure 4j), consistent with the pattern observed for **M1** and indicating that hydroxylation occurred on the dibutylamine moiety. Consequently, **M9a** and **M9b** were characterized as acyl glucuronides of **M1**. As noted with **M1**, the precise position of the hydroxyl group on the butyl chain could not be determined.

Metabolite **M10** eluted at 7.2 min (Figure 2k) and exhibited a protonated molecular ion at *m*/*z* 631.2472 (C_31_H_39_N_2_O_12_). The observed mass shift of **+120 Da** relative to **M0** suggests the combined effects of *N*-dealkylation (**−56 Da**) and glucuronidation (**+176 Da**). In the MS/MS fragmentation analysis, the molecular ion underwent a neutral loss of **176 Da**, generating a fragment ion at *m*/*z* 455.2171, corresponding to **M2** (Figure 3n). Importantly, the observation of the specific product ion at *m*/*z* 530.1666 suggested that the glucuronic acid was conjugated to the acyl moiety. Furthermore, subsequent fragmentation of the *m*/*z* 455.2171 ion yielded a diagnostic ion at *m*/*z* 303.1697 (Figure 4k), consistent with the pattern observed for **M2** and indicating the loss of a butyl group from the amine moiety. Consequently, **M10** was characterized as the acyl glucuronide of **M2** (Table 1).

Metabolite **M11** eluted at 7.6 min (Figure 2l) and exhibited a protonated molecular ion at *m*/*z* 673.2947 (C_34_H_45_N_2_O_12_). The observed mass shift of **+162 Da** relative to **M0** suggests the combined effects of *O*-demethylation (**−14 Da**) and glucuronidation (**+176 Da**). The MS/MS spectrum revealed a characteristic fragment ion at *m*/*z* 497.2640, resulting from the neutral loss of **176 Da**, corresponding to the *O*-demethylated metabolite **M3** (Figure 3o). Subsequent fragmentation of this *m*/*z* 497.2640 ion yielded diagnostic product ions at *m*/*z* 359.2327 and 340.1176 (Figure 4l). Specifically, the fragment ion at *m*/*z* 354.1333 observed in **M0** was replaced by *m*/*z* 340.1176, while the ion at *m*/*z* 359.2327 was retained. This is consistent with the pattern observed for **M3**, confirming that *O*-demethylation occurred on the methoxyphenyl moiety. Consequently, **M11** was characterized as the glucuronide of **M3** (Table 1). Nevertheless, since **M3** contains both a carboxylic acid and a phenolic hydroxyl group derived from *O*-demethylation, the precise site of conjugation—whether it is an acyl glucuronide or an *O*-glucuronide—could not be distinguished by MS/MS analysis.

Metabolite **M12** eluted at 9.4 min (Figure 2m) and exhibited a protonated molecular ion at *m*/*z* 685.2941 (C_35_H_45_N_2_O_12_). The observed mass shift of **+174 Da** relative to **M0** corresponds to the combined effects of dehydrogenation (**−2 Da**) and glucuronidation (**+176 Da**). The MS/MS spectrum revealed a dominant fragment ion at *m*/*z* 509.2639, resulting from the neutral loss of **176 Da**, corresponding to **M4** (Figure 3p). Importantly, the detection of the diagnostic fragment ion at *m*/*z* 530.1666 provided evidence for acyl glucuronidation. Furthermore, subsequent fragmentation of the *m*/*z* 509.2639 ion yielded diagnostic product ions at *m*/*z* 357.2172 and 128.1431 (Figure 4m), consistent with the pattern observed for **M4** and indicating that dehydrogenation occurred on the dibutylamine moiety. Consequently, **M12** was characterized as the acyl glucuronide of **M4** (Table 1). As observed with **M4**, the specific location of the double bond on the alkyl chain remains undefined based on the MS/MS analysis.

Metabolite **M13** eluted at 7.5 min (Figure 2n) and exhibited a protonated molecular ion at *m*/*z* 675.3110 (C_34_H_47_N_2_O_12_). The observed mass shift of **+164 Da** relative to **M0** corresponds to the combined effects of demethylenation (**−12 Da**) and glucuronidation (**+176 Da**). The MS/MS spectrum revealed a dominant fragment ion at *m*/*z* 499.2761, resulting from the neutral loss of **176 Da**, corresponding to **M5** (Figure 3q). Subsequent fragmentation of the *m*/*z* 499.2761 ion yielded diagnostic product ions at *m*/*z* 342.1328 and 164.0697 (Figure 4n), consistent with the pattern observed for **M5** and indicating that demethylenation occurred on the methylenedioxyphenyl moiety. Consequently, **M13** was characterized as the glucuronide conjugate of **M5** (Table 1*).* Given that **M5** possesses a catechol moiety and a carboxylic acid, the exact regiochemistry of glucuronidation remains ambiguous, as the fragmentation pattern did not differentiate between conjugation at the carboxylic acid versus the catechol hydroxyl groups.

Metabolite **M14** eluted at 6.1 min (Figure 2o) and exhibited a protonated molecular ion at *m*/*z* 661.2937 (C_33_H_45_N_2_O_12_). The observed mass shift of **+150 Da** relative to **M0** suggests the combined metabolic modifications of *O*-demethylation (**−14 Da**) and demethylenation (**−12 Da**), followed by glucuronidation (**+176 Da**). In the MS/MS fragmentation analysis, the molecular ion generated a fragment at *m*/*z* 485.2642 via the neutral loss of **176 Da**, corresponding to **M6** (Figure 3r). The subsequent fragmentation pattern of this *m*/*z* 485.2642 ion was identical to that observed for **M6**, specifically featuring the diagnostic ions at *m*/*z* 164.0763 and 107.0493 (Figure 4o), which support the modifications on the methylenedioxyphenyl and methoxyphenyl moieties, respectively. Consequently, **M14** was characterized as the glucuronide conjugate of **M6** (Table 1). Due to the presence of multiple nucleophilic sites on **M6**, including the carboxylic acid and hydroxyl groups generated from concurrent demethylenation and *O*-demethylation, the specific attachment site of the glucuronic acid moiety could not be definitively determined.

### 3.3. Characterization of Atrasentan Reactive Metabolite

Glutathione (GSH) acts as a soft nucleophile that reacts with unstable reactive intermediates to form stable conjugates, thereby facilitating the exploration of potential bioactivation pathways [7,25]. Owing to the transient nature of reactive metabolites, GSH trapping was employed to capture short-lived electrophilic intermediates and elucidate potential bioactivation pathways [26,27].

**Figure 5:**
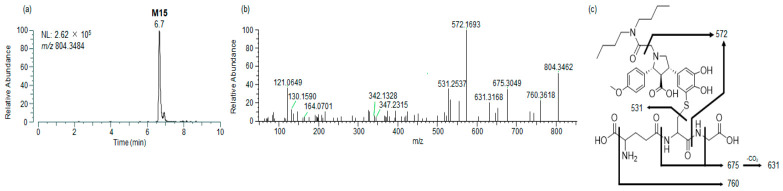
Representative (**a**) extracted ion chromatogram, (**b**) product ion scan mass spectrum and (**c**) fragmentation scheme of reactive metabolites formed during the incubation of atrasentan with human liver microsomes in the presence of NADPH-generating system and glutathione.

**Figure 6:**
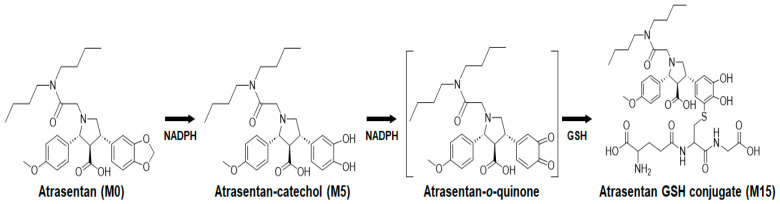
Proposed bioactivation mechanism of the methylenedioxyphenyl group of atrasentan and the formation of glutathione conjugate.

During the incubation of atrasentan with HLMs containing an NADPH-generating system and GSH, the GSH conjugate (**M15**) eluted at 6.7 min (Figure 5a) and exhibited a protonated molecular ion at *m*/*z* 804.3462. In the MS/MS spectrum of **M15**, characteristic fragment ions were observed at *m*/*z* 760.3618, 675.3049, 631.3168, 572.1693, and 531.2537 (Figure 5b,c). Specifically, the fragment ion at *m*/*z* 675.3049 corresponds to the neutral loss of a pyroglutamic acid moiety (**−129 Da**), serving as a characteristic diagnostic ion for GSH conjugation. Additionally, the fragment ion at *m*/*z* 572.1693 resulted from a combined neutral loss of **232 Da** from the precursor ion. This specific mass shift is attributed to the simultaneous loss of the *N,N*-dibutylacetamide moiety (**157 Da**) from the atrasentan skeleton and the glycine residue (**75 Da**) from the GSH tripeptide chain, further supporting the structural integration of the GSH moiety into the parent molecule. More importantly, the fragment ion at *m*/*z* 531.2537 (**−273 Da**) was retained as the catechol core structure containing the sulfur atom from the GSH moiety. This pattern localized the reactive site to the **M5** intermediate, providing direct structural evidence that the methylenedioxyphenyl moiety is oxidized to the **M5** catechol [8,11], which subsequently forms an electrophilic *ortho*-quinone trapped by GSH [23] (Figure 6).

The metabolic bioactivation of drugs into reactive electrophiles is an initiating event in developing adverse drug reactions, particularly DILI [28,29]. While the mechanisms underlying hepatotoxicity are often complex, structural alerts such as the methylenedioxyphenyl moiety in atrasentan are significant [11]. This functional group can be oxidized by P450 enzymes to form carbene intermediates, which undergo further oxidation to generate reactive **M5** or *ortho*-quinones [23,30]. These reactive metabolites can covalently bind to nucleophilic sites on cellular proteins or deplete intracellular GSH pools, mechanisms often associated with cellular toxicity and immune-mediated reactions [31]. However, the formation of reactive metabolites does not inherently indicate hepatotoxicity [26]. The detection of a GSH conjugate supports the formation of an electrophilic intermediate in vitro but does not constitute direct evidence of in vivo hepatotoxicity, widespread oxidative stress, or actual covalent protein binding to vital cellular targets. The in vitro characterization of these electrophilic intermediates serves as a hazard identification tool rather than a definitive clinical risk assessment. Therefore, based solely on these findings, it is not justified to restrict the therapeutic use of atrasentan for managing chronic kidney disease. Instead, these data support clinical recommendations for regular liver function monitoring while taking atrasentan [10,12]. Therefore, further studies such as quantitative assessment of covalent binding to hepatic proteins are warranted to clarify the relationship between M15 and atrasentan-induced hepatotoxicity.

### 3.4. Characterization of P450 Isoforms Involved in Atrasentan Metabolism

To comprehensively assess the metabolic characteristics and potential bioactivation pathways of atrasentan, reaction phenotyping was performed using human recombinant P450 isoforms. Preliminary time-course evaluations in HLMs up to 60 min demonstrated that the formation of the primary metabolites **M2**, **M3**, and **M5** proceeded linearly at both 10 and 50 µM substrate concentrations (Figure S1). This linear kinetic profile indicates that the enzymatic capacity was not saturated under the tested conditions, ensuring the quantitative reliability of the metabolic profiling.

**Figure 7:**
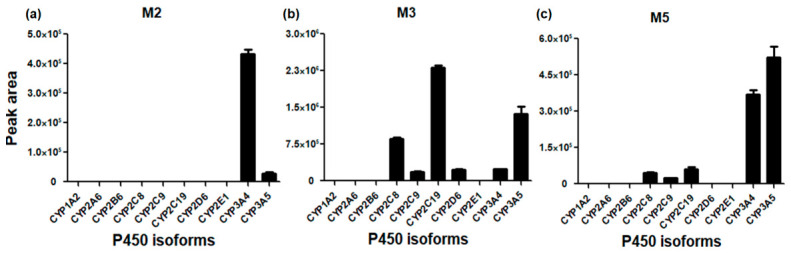
Formation of (**a**) **M2**, *N*-desbutyl atrasentan, (**b**) **M3**, *O*-desmethyl atrasentan, and (**c**) **M5**, atrasentan-catechol, by human recombinant P450 isoforms. The metabolic activities were evaluated by measuring the peak areas of the metabolites generated after incubation of atrasentan with a panel of human recombinant P450 isoforms. The incubation was performed for 30 min. Background signals from control samples containing microsomes deactivated by heating at 100 °C for 30 min were subtracted. The bars represent the mean ± standard deviation (SD) of independent triplicates.

The phenotypic profiling revealed distinct enzymatic dependencies linked to specific bioactivation routes. The formation of **M2** was almost exclusively catalyzed by CYP3A4, accounting for nearly 100% of the relative contribution in the scaled data (Figure 7a). This absolute reliance indicates a highly specific enzyme-substrate affinity for this particular route, demonstrating that the metabolic clearance of atrasentan through *N*-dealkylation is driven by CYP3A4. In contrast, the formation of M3 was mediated by a diverse set of CYP enzymes, with CYP2C19 and the CYP3A subfamily emerging as the primary contributors (Figure 7b). Minor participation was also observed from CYP2C8, CYP2C9, and CYP2D6.

The formation of **M5** was predominantly mediated by the CYP3A subfamily, with CYP3A4 acting as the major contributor (Figure 7c). Crucially, structural elucidation characterized M5 as a catechol intermediate, a well-established precursor to putative reactive quinone species. This significant reliance on CYP3A for the generation of the reactive catechol intermediate provides critical mechanistic insights into atrasentan bioactivation. Notably, the metabolic trends and P450 contribution profiles delineated in this study closely mirror the previously reported disposition of atrasentan [32]. This strong consensus underscores the reliability of our experimental framework, confirming that CYP3A4 drives both the primary metabolic clearance of atrasentan and its bioactivation to the **M5** catechol intermediate.

### 3.5. Profiling of Atrasentan Metabolites Using Feature-Based Molecular Networking

**Figure 8:**
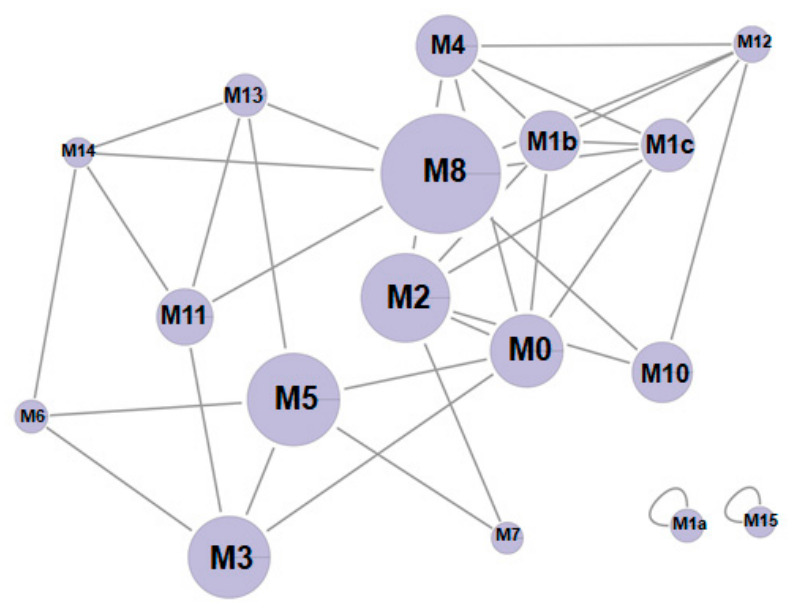
Representative molecular network of MS/MS spectra from the liquid chromatography–high resolution mass spectrometry analysis of human liver microsomal samples of atrasentan (**M0**). Table 1 lists the information on each metabolite. The molecular network result can be visualized directly on GNPS using the following links: https://gnps.ucsd.edu/ProteoSAFe/status.jsp?task=2bf9779c95ff406bbc606dc64ba7060b, accessed on 19 November 2025.

FBMN analysis was used to visualize the global metabolic profile and spectral relationships between atrasentan and its metabolites [33]. The molecular network was constructed based on MS/MS spectral similarity (Figure 8), where nodes represent detected molecular features and edges denote a cosine similarity score exceeding 0.6 [15,34]. **M0** served as the central hub within the major cluster, showing high spectral similarity with its metabolites, which supports the structural characterization from the fragmentation patterns.

In phase I metabolism, **M0** acted as the primary central node, directly linking to the initial metabolites (**M1**–**M5**). Owing to the conservation of the core scaffold despite metabolic modifications, phase I metabolites formed a strongly connected network with the parent drug. Furthermore, **M5** acted as a secondary node, showing spectral links to downstream metabolites (**M6**, **M7**, and **M13**). This visualizes the sequential metabolic pathway involving the oxidation of the methylenedioxyphenyl moiety.

In phase II metabolism, **M8** acted as a secondary node, linking atrasentan to other glucuronide conjugates. Owing to the characteristic neutral loss of glucuronic acid (**176 Da**) and shared fragment ions derived from the glucuronic acid moiety, the glucuronidated metabolites (**M10**–**M14**) formed a distinct subcluster [35]. Simultaneously, through conserved core scaffold fragments, they maintained structural connections with their respective phase I precursors (**M2**–**M6**) as well as the primary phase II node, **M8**. In contrast, **M1a** and **M15** appeared as isolated nodes, distinct from the main cluster. As demonstrated in the bioactivation study, this isolation of **M15** is likely due to significant alterations in fragmentation patterns caused by GSH conjugation and complex structural modifications. Consequently, the FBMN analysis provided a comprehensive overview of atrasentan metabolism, visually confirming its extensive bioactivation in HLMs.

However, it is important to acknowledge a technical limitation of the FBMN approach used in this study. Since molecular networking relies heavily on MS/MS spectral similarity, metabolites with significant structural modifications may fail to cluster with the parent drug [36,37]. A notable example in our results is the glutathione conjugate M15, which appeared as an isolated node (Figure 8). The conjugation of the bulky GSH moiety drastically altered the fragmentation pattern compared to atrasentan (**M0**) [38], resulting in a cosine similarity score below the threshold of 0.6. This isolation highlights that while FBMN is a powerful tool for grouping structurally related compounds, it may generate false negatives for complex conjugates. Therefore, manual inspection and structural analysis remain essential to prevent overlooking metabolically significant but spectrally distinct compounds. Furthermore, while the current experimental conditions captured a comprehensive metabolic profile of atrasentan, the possibility that some minor or highly transient metabolites were not detected cannot be entirely ruled out. Factors such as ionization selectivity in positive ESI mode [39], the inherent instability of certain reactive intermediates [38], or the inherent clustering and filtering parameters of the FBMN workflow could contribute to this [36]. Accordingly, future investigations integrating complementary analytical platforms or orthogonal ionization techniques are warranted to comprehensively delineate this intricate metabolic landscape.

## 4. Conclusions

In this study, the in vitro metabolism of atrasentan by HLMs was investigated using FBMN combined with LC-HRMS. Based on accurate mass measurements and distinctive fragment ion patterns, the metabolite structures were systematically characterized, using the fragmentation of atrasentan (**M0**) as a reference.

**Figure 9:**
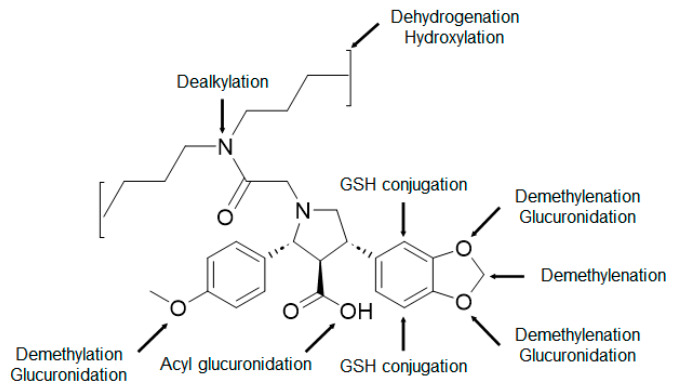
Chemical structure of atrasentan showing the sites of glutathione conjugation and phase I and II bioactivation pathways.

Eighteen metabolites (**M1**–**M14**), along with GSH conjugate **M15**, were characterized. The results showed that atrasentan underwent extensive bioactivation through major phase I and II metabolic pathways: (1) hydroxylation, (2) *N*-dealkylation, (3) *O*-demethylation, (4) dehydrogenation, (5) demethylenation, and (6) glucuronidation. Among the characterized metabolites, **M5** was the predominant phase I metabolite, while **M8** was the major phase II metabolite, providing a comprehensive overview of the metabolic landscape (Figure 9). The detection of **M15** indicates the potential for bioactivation. This finding provides direct evidence that the methylenedioxyphenyl moiety of atrasentan can be oxidized to a reactive *ortho*-quinone intermediate, which was subsequently trapped by GSH [4]. However, it is important to note that **M15** appeared as an isolated node in the FBMN analysis. The GSH moiety significantly altered the fragmentation pattern, resulting in low spectral similarity to the parent drug. This highlights a limitation of FBMN, where structurally distinct conjugates may fail to cluster with the core scaffold, necessitating complementary manual inspection to avoid false negatives [36].

**Figure 10:**
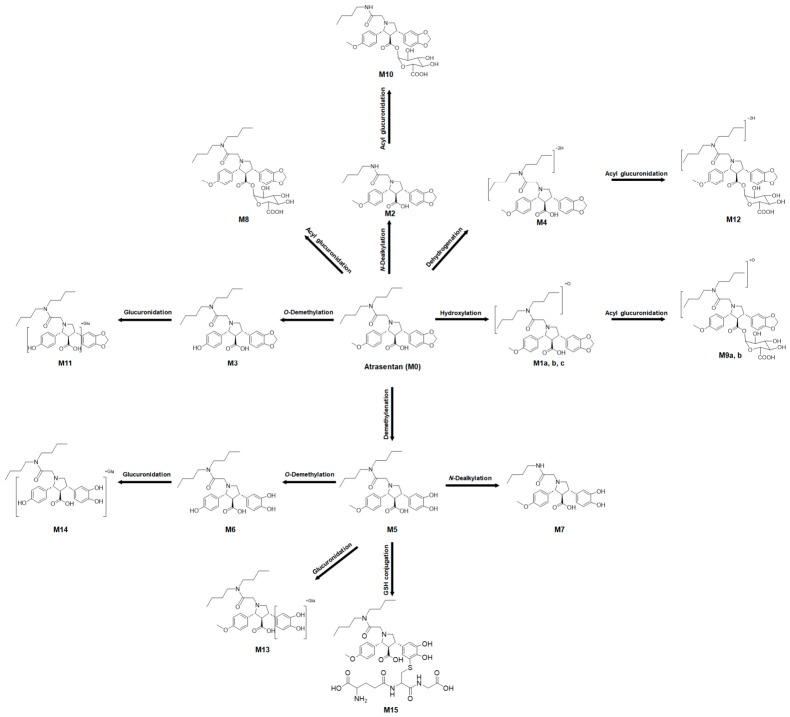
Proposed metabolic pathway of atrasentan in human liver microsomes. Atrasentan (**M0**) underwent extensive hydroxylation, dealkylation, demethylation, dehydrogenation, and glucuronidation reactions. **M5** and **M8** are identified as major metabolites, with **M5** further metabolized via **M6** or **M7**. **M5** and **M6** were subsequently glucuronidated to form **M13** and **M14**, respectively. Additionally, **M15**, derived from **M5**, indicates a bioactivation pathway involving a reactive *ortho*-quinone intermediate. **Abbreviations**: **M0**, atrasentan; **M5**, catechol intermediate; **M8**, acyl glucuronide; **M6**, *O*-demethylation metabolite; **M7**, *N*-dealkylation metabolite; **M15**, glutathione conjugate.

In conclusion, this study elucidates the metabolic profile of atrasentan in HLMs, underscoring the precarious equilibrium between glucuronidation and P450-catalyzed bioactivation pathways (Figure 10). Reaction phenotyping revealed that CYP3A4 and CYP3A5 almost catalyze the bioactivation pathway leading to the catechol intermediate (**M5**). While the in vitro studies do not warrant restricting its clinical application, they emphasize the value of regular liver function monitoring in clinical settings [28,29]. Furthermore, the need for further in vivo studies to establish the causal link between these reactive metabolites and potential hepatotoxicity.

## Figures and Tables

**Table 1:** Summary of atrasentan metabolites characterized in human liver microsomal incubation samples.

No.	Assignment	t_R_ (min)	[M+H]^+^		Mass Error (ppm)	Formula (Neutral)	Fragment ions (*m*/*z*)	Peak Intensity ^1^
			Measured	Theoretical				
M0	Atrasentan	9.3	511.2802	511.2803	−0.20	C_29_H_38_N_2_O_6_	359.2324, 354.1333, 176.0706, 135.0438, 130.1590, 121.0649	
M1a	Hydroxylation	7.0	527.2745	527.2752	−1.33	C_29_H_38_N_2_O_7_	**375.2272**, 354.1328, 176.0703, **146.1539**, 135.0438, 121.0647	++
M1b	Hydroxylation	7.4	527.2744	527.2752	−1.52	C_29_H_38_N_2_O_7_	**375.2271**, 354.1328, 176.0703, **146.1536**, 135.0438, 121.0647	+++
M1c	Hydroxylation	8.0	527.2738	527.2752	−2.66	C_29_H_38_N_2_O_7_	**375.2269**, 354.1328, 176.0703, **146.1538**, 135.0439, 121.0648	+++
M2	*N*-Dealkylation	8.2	455.2164	455.2177	−2.86	C_25_H_30_N_2_O_6_	354.1335, **303.1696**, 176.0704, 135.0438, 121.0648, **74.0970**,	+++
M3	*O*-Demethylation	8.4	497.2641	497.2646	−1.01	C_28_H_36_N_2_O_6_	359.2323, **340.1173**, 176.0704, 135.0438, **132.0444**, 130.1589, **107.0493**	+++
M4	Dehydrogenation	10.0	509.2638	509.2646	−1.57	C_29_H_36_N_2_O_6_	**357.2165**, 354.1331, 176.0704, 135.0439, **128.1434**, 121.0649	+++
M5	Demethylenation	8.2	499.2799	499.2803	−0.80	C_28_H_38_N_2_O_6_	**347.2318**, **342.1331**, **164.0706**, 130.1589, **123.0439**, 121.0648	++++
M6	Demethylenation + *O*-Demethylation	6.9	485.2622	485.2646	−4.95	C_27_H_36_N_2_O_6_	**328.1172**, **164.0703**, 130.1591, **123.0440**, **107.0493**	++
M7	Demethylenation + *N*-Dealkylation	5.4	443.2183	443.2177	1.35	C_24_H_30_N_2_O_6_	**342.1332**, **291.1697**, **164.0704**, **123.0442**, 121.0649, **74.0970**	++
M8	Acyl glucuronidation	8.7	687.3118	687.3123	−0.73	C_35_H_46_N_2_O_12_	**530.1647**, **511.2800**, 359.2323, 354.1331, 176.0707, 135.0440, 130.1591, 121.0649	++++
M9a	Hydroxylation + Acyl glucuronidation	6.3	703.3057	703.3073	−2.27	C_35_H_46_N_2_O_13_	**530.1663**, **527.2742**, **375.2269**, 354.1337, 176.0703, **146.1538**, 135.0440, 121.0649	+
M9b	Hydroxylation + Acyl glucuronidation	6.9	703.3070	703.3073	−0.43	C_35_H_46_N_2_O_13_	**530.1650**, **527.2750**, **375.2286**, 354.1334, 176.0703, **146.1538**, 135.0440, 121.0649	+
M10	*N*-Dealkylation + Acyl glucuronidation	7.2	631.2472	631.2498	−4.12	C_31_H_38_N_2_O_12_	**530.1666, 455.2171**, 354.1337, **303.1697**, 176.0703, 135.0440, 121.0649, **74.0968**	+++
M11	*O*-Demethylation + Glucuronidation	7.6	673.2947	673.2967	−2.97	C_34_H_44_N_2_O_12_	**516.1491**, **497.2640**, 359.2327, **340.1176**, 176.0704, 135.0440, 130.1594, **107.0499**	+++
M12	Dehydrogenation + Acyl glucuronidation	9.4	685.2941	685.2967	−3.79	C_35_H_44_N_2_O_12_	**530.1647**, **509.2639**, **357.2172**, 354.1331, 176.0703, 135.0440, **128.1431**, 121.0651	++
M13	Demethylenation + Glucuronidation	7.5	675.3110	675.3124	−2.07	C_34_H_46_N_2_O_12_	**523.2631**, **518.1651**, **499.2761**, **342.1328**, **164.0697**, 130.1590, 121.0648	+++
M14	Demethylenation + *O*-Demethylation + Glucuronidation	6.1	661.2937	661.2967	−4.54	C_33_H_44_N_2_O_12_	**504.1499**, **485.2642**, **328.1175**, **164.0703**, 130.1590, **123.0440**, 107.0493	++
M15	Demethylenation + GSH conjugation	6.7	804.3462	804.3484	−2.74	C_38_H_53_N_5_O_12_S	760.3618, 675.3049, 631.3168, 572.1693, 531.2537, 347.2315, 342.1328, 164.0701, 130.1590, 121.0649	++

^1^ ++++ detected >10^7^; +++ detected > 10^6^; ++ detected > 10^5^; + detected > 10^4^. Note: Bold values indicate the specific fragment ions utilized for the structural identification of the corresponding metabolites.

## Data Availability

The original contributions presented in this study are included in the article and Supplementary Materials. Further inquiries can be directed to the corresponding authors.

## Supplementary Materials

The following supporting information can be downloaded at: https://www.mdpi.com/article/10.3390/pharmaceutics18060731/s1, Figure S1: Time-dependent formation of atrasentan metabolites (a) M2, *N*-desbutyl atrasentan, (b) M3, *O*-desmethyl atrasentan, and (c) M5, atrasentan-catechol, in human liver microsomes at atrasentan 10 and 50 μM.

## Abbreviations

SERA	Selective endothelin A receptor antagonist
DILI	Drug-induced liver injury
P450	Cytochrome P450
GSH	Glutathione
HLMs	Human liver microsomes
NADPH	Nicotinamide adenine dinucleotide phosphate
UDPGA	Uridine diphosphate glucuronic acid
LC-HRMS	Liquid chromatography–high resolution mass spectrometry
FBMN	Feature-Based Molecular Networking
G6P	Glucose-6-phosphate
G6PDH	Glucose-6-phosphate dehydrogenase
NADP^+^	Nicotinamide adenine dinucleotide phosphate
MgCl_2_	Magnesium chloride
ESI	Electrospray ionization
MS/MS	Tandem mass spectrometry
ddMS^2^	Data-dependent MS/MS
