# Supplementary material for: Characterization of Atrasentan Metabolic Pathway in Human Liver Microsomes Using Feature-Based Molecular Networking
Source: Pharmaceutics. 2026 Jun 13;18(6):731. doi: 10.3390/pharmaceutics18060731 (PMC13306606; doi:10.3390/pharmaceutics18060731)
Supplement: Supplementary file 1 [file pharmaceutics-18-00731-s001.zip › pharmaceutics-4327294-supplementary.pdf]

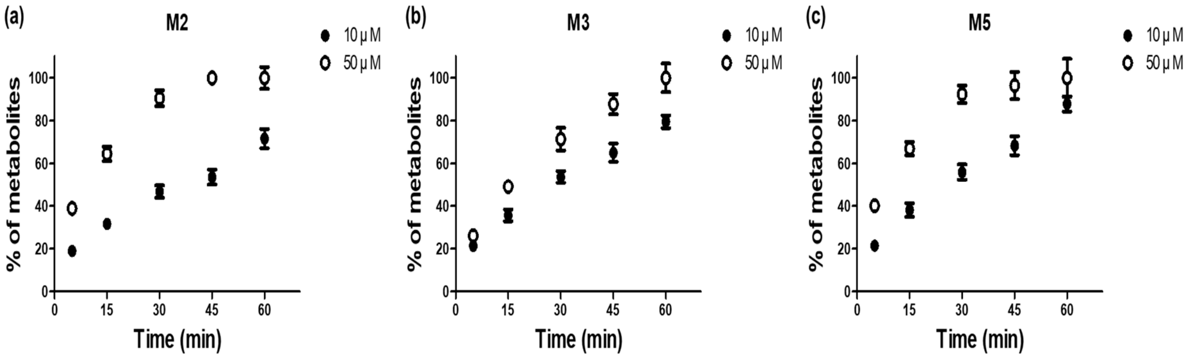

Figure S1. Time-dependent formation of atrasentan metabolites (a) **M2**, *N*-desbutyl atrasentan, (b) **M3**, *O*-desmethyl atrasentan, and (c) **M5**, atrasentan-catechol, in human liver microsomes at atrasentan 10 and 50 μM. The metabolic activities were evaluated by determining the relative percentage (% of metabolites) generated after the incubation of atrasentan with human liver microsomes at the indicated time points up to 60 minutes. Background signals from control samples containing microsomes deactivated by heating at 100 °C for 30 minutes were subtracted. Data points represent the mean ± standard deviation (SD) of independent triplicates.
